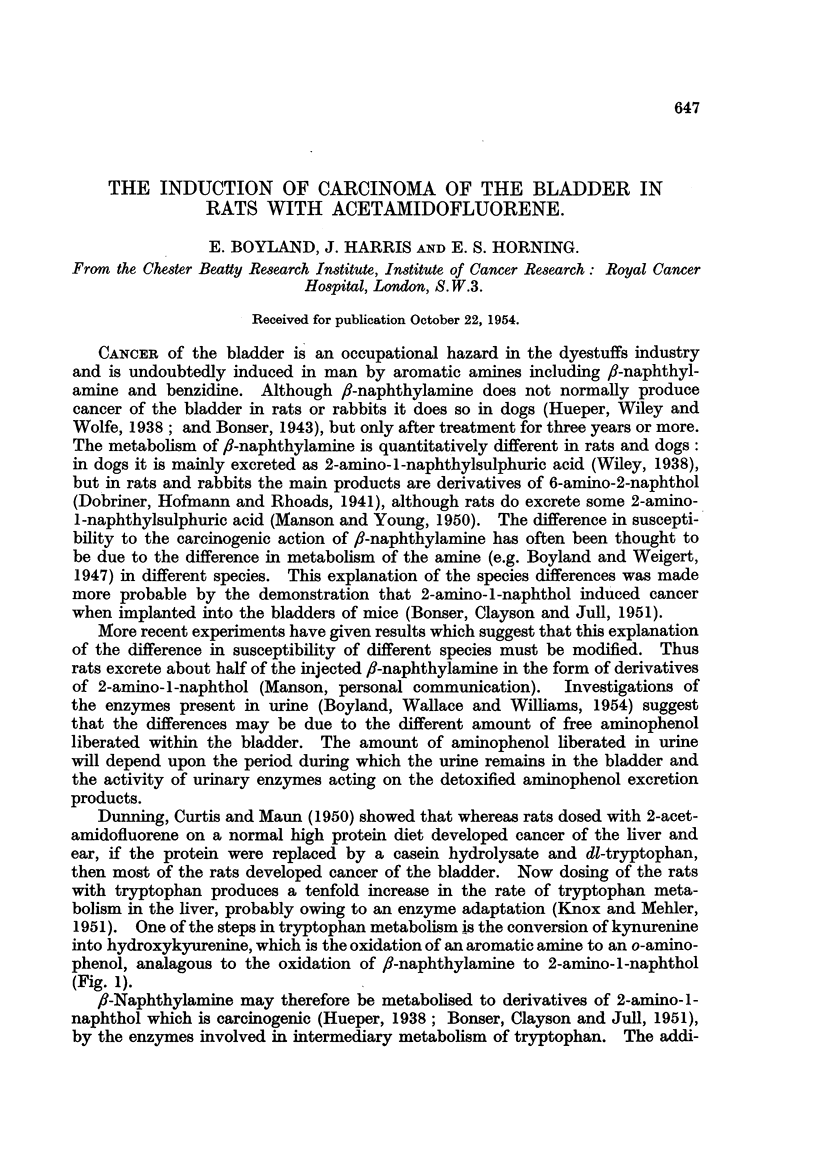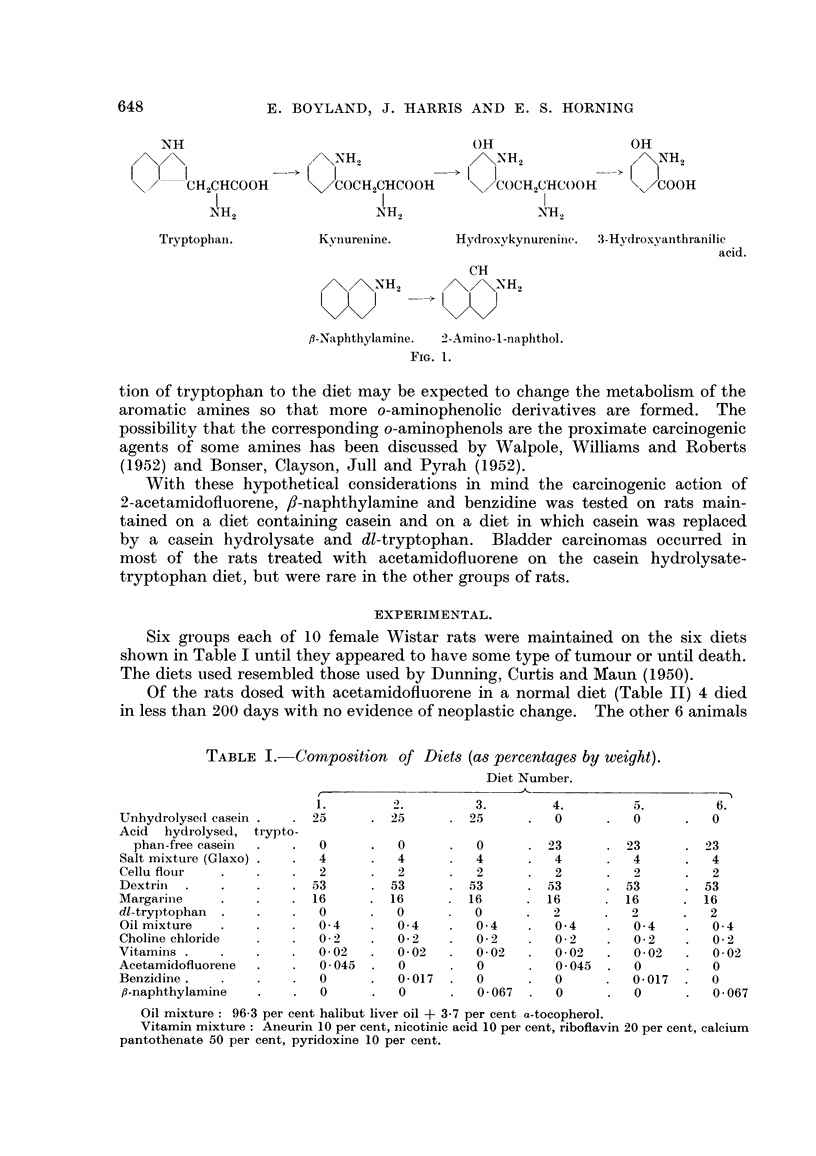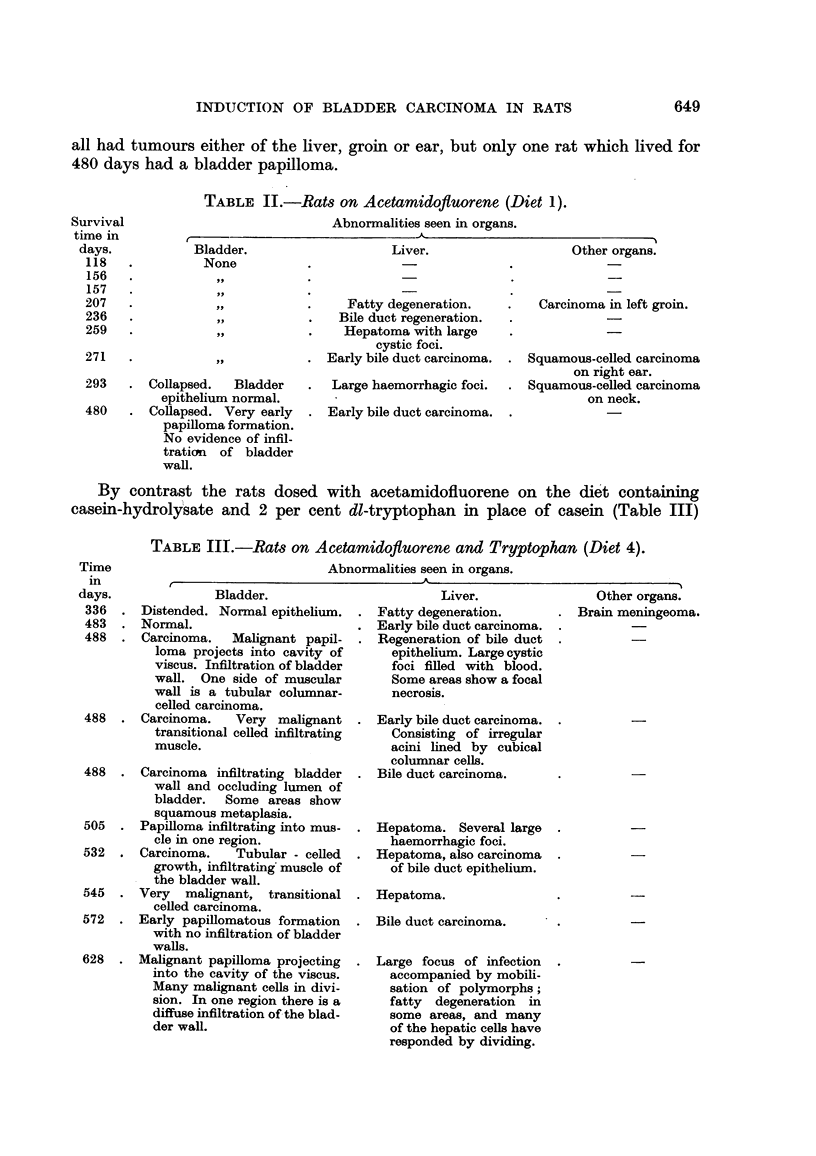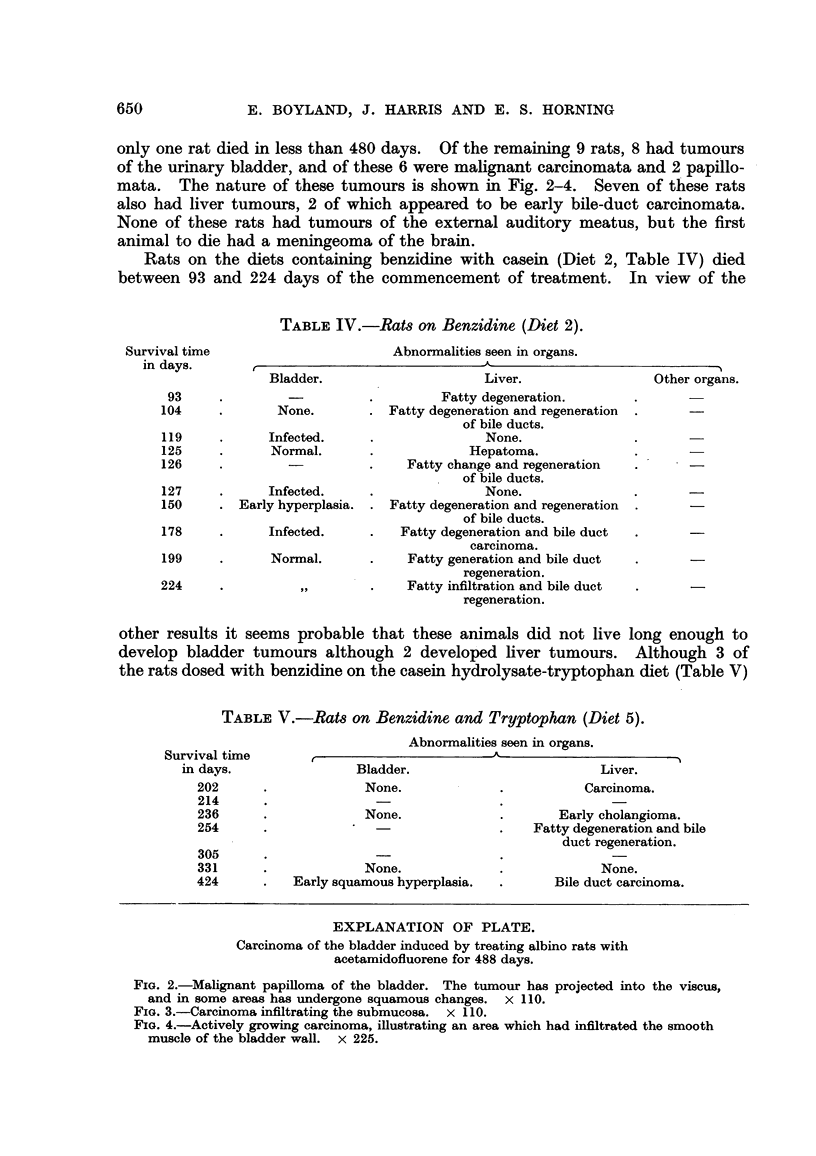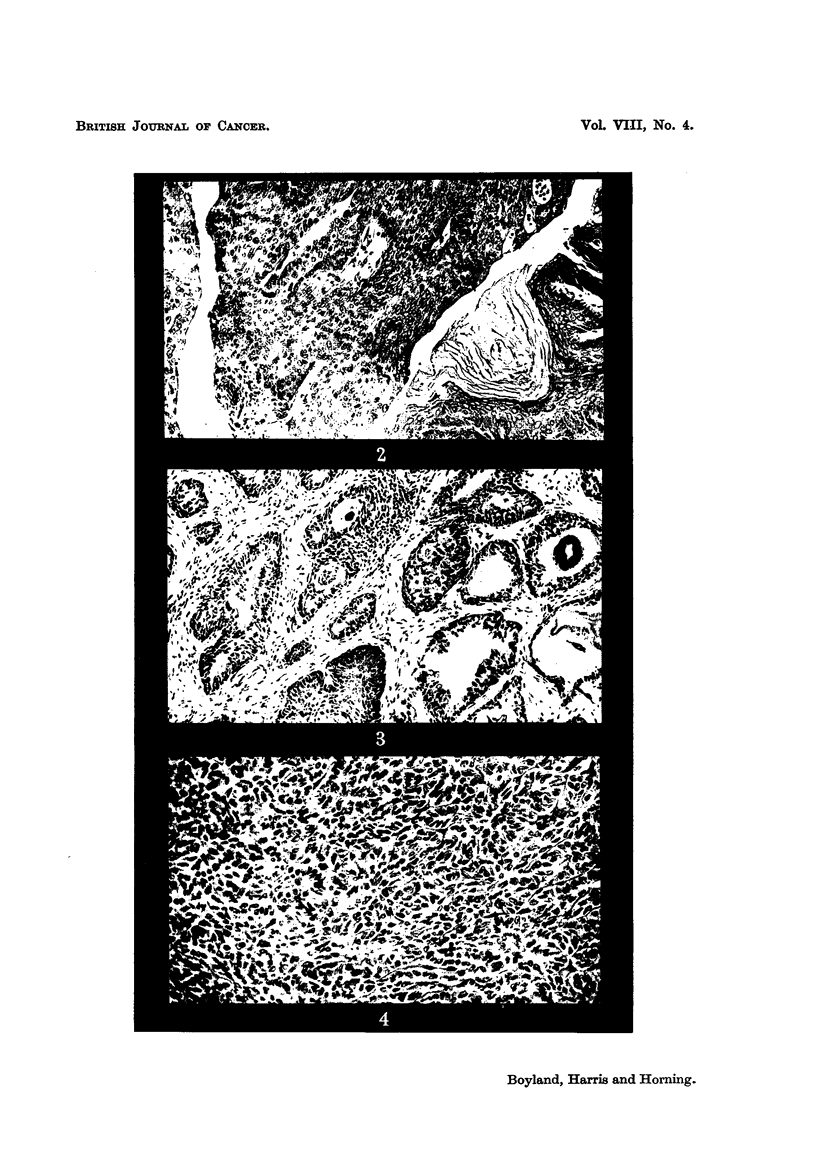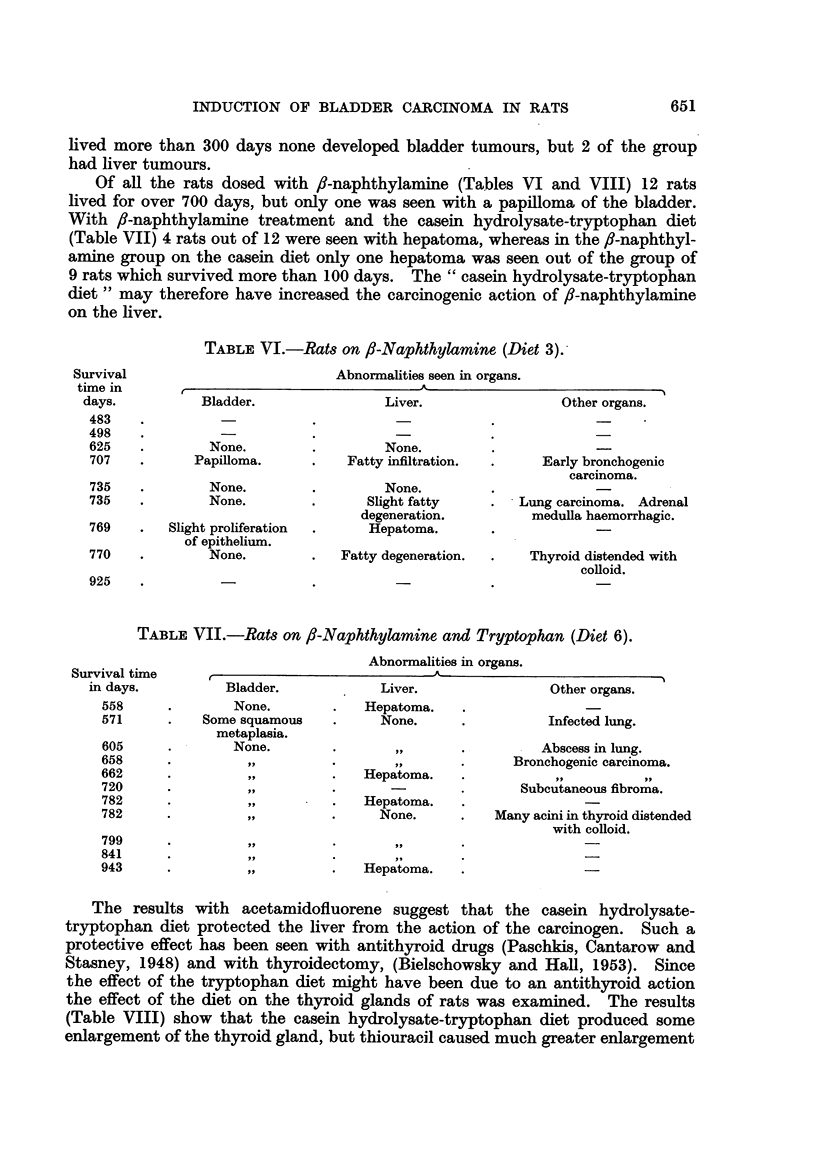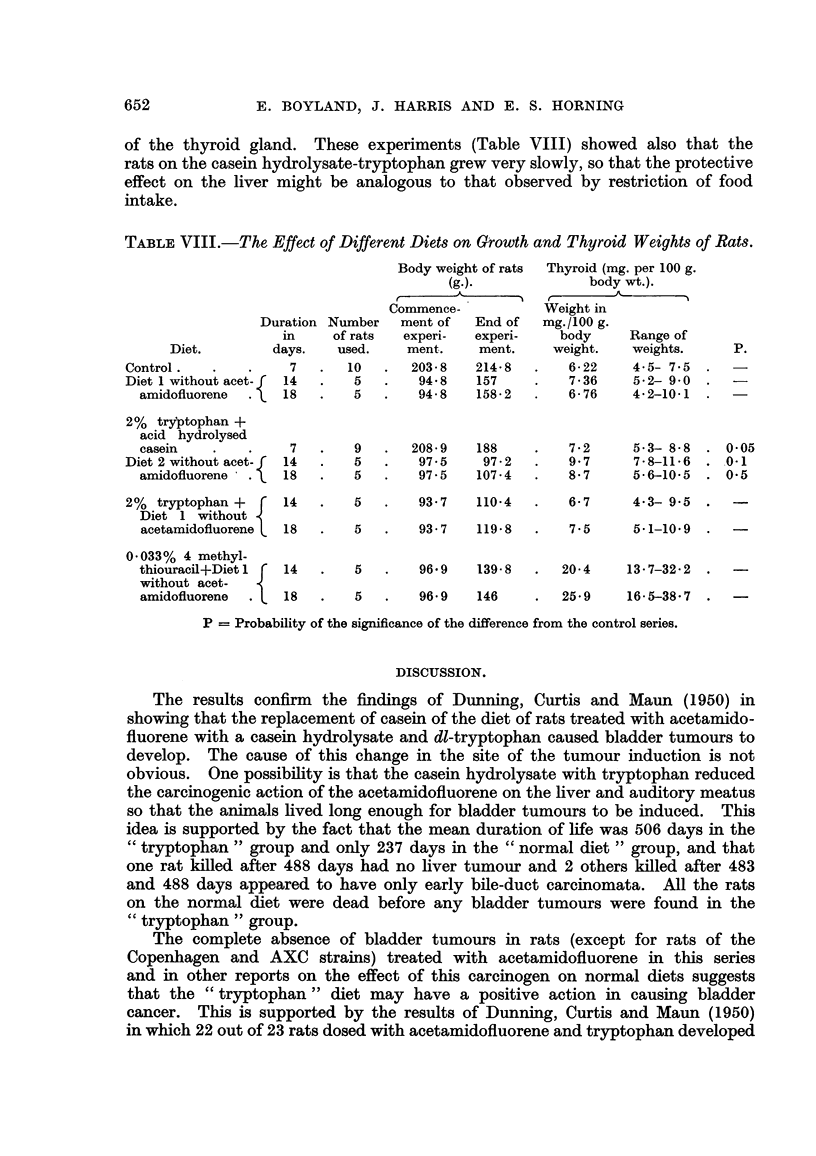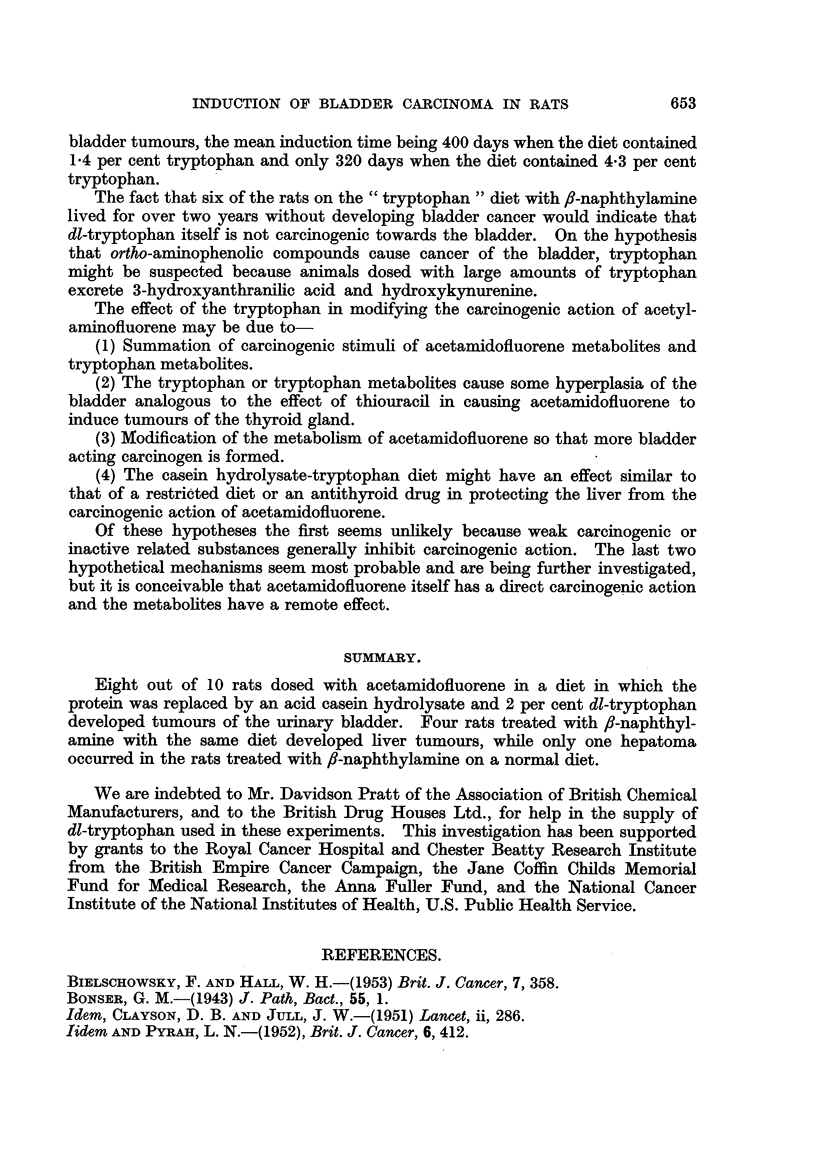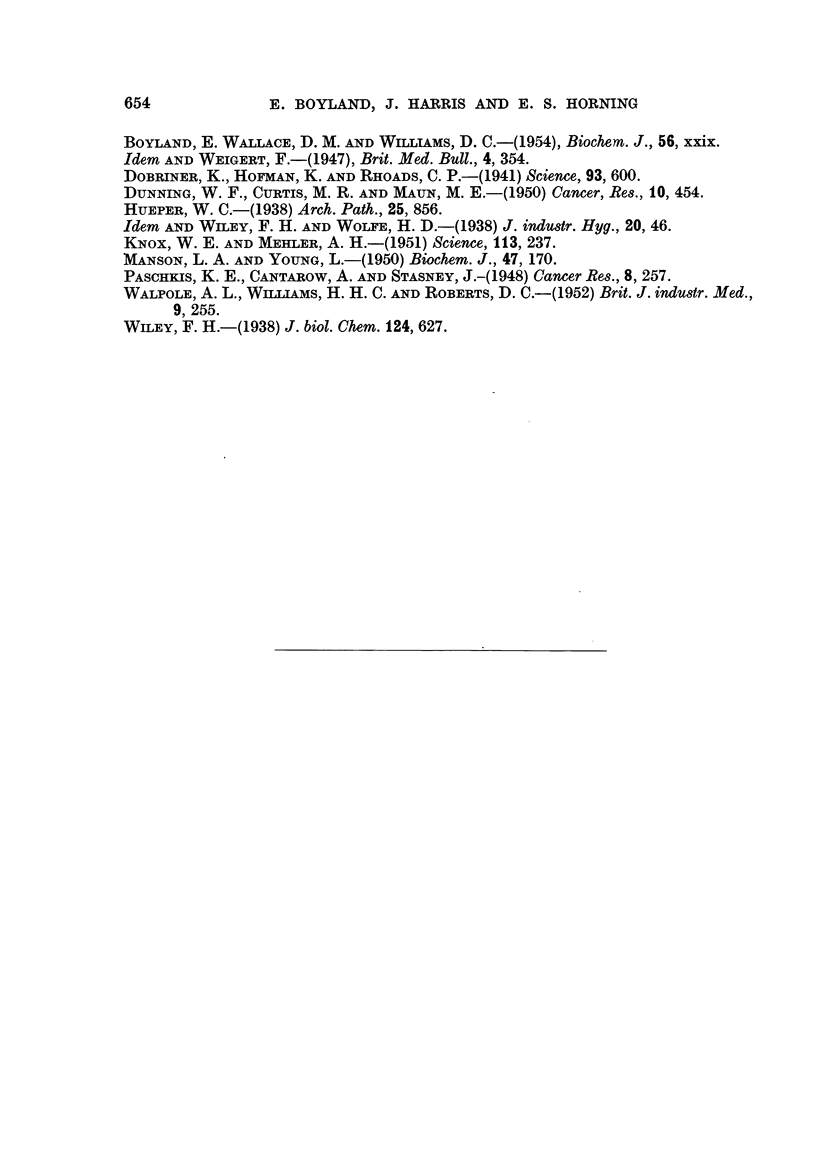# The Induction of Carcinoma of the Bladder in Rats with Acetamidofluorene

**DOI:** 10.1038/bjc.1954.70

**Published:** 1954-12

**Authors:** E. Boyland, J. Harris, E. S. Horning

## Abstract

**Images:**


### THE INDUCTION OF CARCINOMA OF THE BLADDER IN RATS WITH ACETAMIDOFLUORENE.

E. BOYLAND, J. HARRIS AND E. S. HORNING.

*From the Chester Beatty Research Institute, Institute of Cancer Research : Royal Cancer Hospital, London, S.W.3.*

Received for publication October 22, 1954.

CANCER of the bladder is an occupational hazard in the dyestuffs industry and is undoubtedly induced in man by aromatic amines including $\beta$-naphthylamine and benzidine. Although $\beta$-naphthylamine does not normally produce cancer of the bladder in rats or rabbits it does so in dogs (Hueper, Wiley and Wolfe, 1938 ; and Bonser, 1943), but only after treatment for three years or more. The metabolism of $\beta$-naphthylamine is quantitatively different in rats and dogs : in dogs it is mainly excreted as 2-amino-1-naphthylsulphuric acid (Wiley, 1938), but in rats and rabbits the main products are derivatives of 6-amino-2-naphthol (Dobriner, Hofmann and Rhoads, 1941), although rats do excrete some 2-amino-1-naphthylsulphuric acid (Manson and Young, 1950). The difference in susceptibility to the carcinogenic action of $\beta$-naphthylamine has often been thought to be due to the difference in metabolism of the amine (e.g. Boyland and Weigert, 1947) in different species. This explanation of the species differences was made more probable by the demonstration that 2-amino-1-naphthol induced cancer when implanted into the bladders of mice (Bonser, Clayson and Jull, 1951).

More recent experiments have given results which suggest that this explanation of the difference in susceptibility of different species must be modified. Thus rats excrete about half of the injected $\beta$-naphthylamine in the form of derivatives of 2-amino-1-naphthol (Manson, personal communication). Investigations of the enzymes present in urine (Boyland, Wallace and Williams, 1954) suggest that the differences may be due to the different amount of free aminophenol liberated within the bladder. The amount of aminophenol liberated in urine will depend upon the period during which the urine remains in the bladder and the activity of urinary enzymes acting on the detoxified aminophenol excretion products.

Dunning, Curtis and Maun (1950) showed that whereas rats dosed with 2-acetamidofluorene on a normal high protein diet developed cancer of the liver and ear, if the protein were replaced by a casein hydrolysate and *dl*-tryptophan, then most of the rats developed cancer of the bladder. Now dosing of the rats with tryptophan produces a tenfold increase in the rate of tryptophan metabolism in the liver, probably owing to an enzyme adaptation (Knox and Mehler, 1951). One of the steps in tryptophan metabolism is the conversion of kynurenine into hydroxykyurenine, which is the oxidation of an aromatic amine to an *o*-aminophenol, analagous to the oxidation of $\beta$-naphthylamine to 2-amino-1-naphthol (Fig. 1).

$\beta$-Naphthylamine may therefore be metabolised to derivatives of 2-amino-1-naphthol which is carcinogenic (Hueper, 1938 ; Bonser, Clayson and Jull, 1951), by the enzymes involved in intermediary metabolism of tryptophan. The addi-

Tryptophan. Kynurenine. Hydroxykynurenine. 3-Hydroxyanthranilic acid.

$\beta$-Naphthylamine. 2-Amino-1-naphthol.

FIG. 1.

tion of tryptophan to the diet may be expected to change the metabolism of the aromatic amines so that more *o*-aminophenolic derivatives are formed. The possibility that the corresponding *o*-aminophenols are the proximate carcinogenic agents of some amines has been discussed by Walpole, Williams and Roberts (1952) and Bonser, Clayson, Jull and Pyrah (1952).

With these hypothetical considerations in mind the carcinogenic action of 2-acetamidofluorene, $\beta$-naphthylamine and benzidine was tested on rats maintained on a diet containing casein and on a diet in which casein was replaced by a casein hydrolysate and *dl*-tryptophan. Bladder carcinomas occurred in most of the rats treated with acetamidofluorene on the casein hydrolysate-tryptophan diet, but were rare in the other groups of rats.

### EXPERIMENTAL.

Six groups each of 10 female Wistar rats were maintained on the six diets shown in Table I until they appeared to have some type of tumour or until death. The diets used resembled those used by Dunning, Curtis and Maun (1950).

Of the rats dosed with acetamidofluorene in a normal diet (Table II) 4 died in less than 200 days with no evidence of neoplastic change. The other 6 animals

TABLE I.—*Composition of Diets (as percentages by weight).*

| | Diet Number. | | | | | |
|---|---|---|---|---|---|---|
| | 1. | 2. | 3. | 4. | 5. | 6. |
| Unhydrolysed casein | 25 | 25 | 25 | 0 | 0 | 0 |
| Acid hydrolysed, tryptophan-free casein | 0 | 0 | 0 | 23 | 23 | 23 |
| Salt mixture (Glaxo) | 4 | 4 | 4 | 4 | 4 | 4 |
| Cellu flour | 2 | 2 | 2 | 2 | 2 | 2 |
| Dextrin | 53 | 53 | 53 | 53 | 53 | 53 |
| Margarine | 16 | 16 | 16 | 16 | 16 | 16 |
| *dl*-tryptophan | 0 | 0 | 0 | 2 | 2 | 2 |
| Oil mixture | 0·4 | 0·4 | 0·4 | 0·4 | 0·4 | 0·4 |
| Choline chloride | 0·2 | 0·2 | 0·2 | 0·2 | 0·2 | 0·2 |
| Vitamins | 0·02 | 0·02 | 0·02 | 0·02 | 0·02 | 0·02 |
| Acetamidofluorene | 0·045 | 0 | 0 | 0·045 | 0 | 0 |
| Benzidine | 0 | 0·017 | 0 | 0 | 0·017 | 0 |
| $\beta$-naphthylamine | 0 | 0 | 0·067 | 0 | 0 | 0·067 |

Oil mixture : 96·3 per cent halibut liver oil + 3·7 per cent $\alpha$-tocopherol.

Vitamin mixture : Aneurin 10 per cent, nicotinic acid 10 per cent, riboflavin 20 per cent, calcium pantothenate 50 per cent, pyridoxine 10 per cent.

all had tumours either of the liver, groin or ear, but only one rat which lived for 480 days had a bladder papilloma.

TABLE II.—*Rats on Acetamidofluorene* (*Diet* 1).

| Survival time in days. | Abnormalities seen in organs. Bladder. | Liver. | Other organs. |
|---|---|---|---|
| 118 | None | — | — |
| 156 | ,, | — | — |
| 157 | ,, | — | — |
| 207 | ,, | Fatty degeneration. | Carcinoma in left groin. |
| 236 | ,, | Bile duct regeneration. | — |
| 259 | ,, | Hepatoma with large cystic foci. | — |
| 271 | ,, | Early bile duct carcinoma. | Squamous-celled carcinoma on right ear. |
| 293 | Collapsed. Bladder epithelium normal. | Large haemorrhagic foci. | Squamous-celled carcinoma on neck. |
| 480 | Collapsed. Very early papilloma formation. No evidence of infiltration of bladder wall. | Early bile duct carcinoma. | — |

By contrast the rats dosed with acetamidofluorene on the diet containing casein-hydrolysate and 2 per cent *dl*-tryptophan in place of casein (Table III)

TABLE III.—*Rats on Acetamidofluorene and Tryptophan* (*Diet* 4).

| Time in days. | Abnormalities seen in organs. Bladder. | Liver. | Other organs. |
|---|---|---|---|
| 336 | Distended. Normal epithelium. | Fatty degeneration. | Brain meningeoma. |
| 483 | Normal. | Early bile duct carcinoma. | — |
| 488 | Carcinoma. Malignant papilloma projects into cavity of viscus. Infiltration of bladder wall. One side of muscular wall is a tubular columnar-celled carcinoma. | Regeneration of bile duct epithelium. Large cystic foci filled with blood. Some areas show a focal necrosis. | — |
| 488 | Carcinoma. Very malignant transitional celled infiltrating muscle. | Early bile duct carcinoma. Consisting of irregular acini lined by cubical columnar cells. | — |
| 488 | Carcinoma infiltrating bladder wall and occluding lumen of bladder. Some areas show squamous metaplasia. | Bile duct carcinoma. | — |
| 505 | Papilloma infiltrating into muscle in one region. | Hepatoma. Several large haemorrhagic foci. | — |
| 532 | Carcinoma. Tubular - celled growth, infiltrating muscle of the bladder wall. | Hepatoma, also carcinoma of bile duct epithelium. | — |
| 545 | Very malignant, transitional celled carcinoma. | Hepatoma. | — |
| 572 | Early papillomatous formation with no infiltration of bladder walls. | Bile duct carcinoma. | — |
| 628 | Malignant papilloma projecting into the cavity of the viscus. Many malignant cells in division. In one region there is a diffuse infiltration of the bladder wall. | Large focus of infection accompanied by mobilisation of polymorphs; fatty degeneration in some areas, and many of the hepatic cells have responded by dividing. | — |

only one rat died in less than 480 days. Of the remaining 9 rats, 8 had tumours of the urinary bladder, and of these 6 were malignant carcinomata and 2 papillomata. The nature of these tumours is shown in Fig. 2–4. Seven of these rats also had liver tumours, 2 of which appeared to be early bile-duct carcinomata. None of these rats had tumours of the external auditory meatus, but the first animal to die had a meningeoma of the brain.

Rats on the diets containing benzidine with casein (Diet 2, Table IV) died between 93 and 224 days of the commencement of treatment. In view of the other results it seems probable that these animals did not live long enough to develop bladder tumours although 2 developed liver tumours. Although 3 of the rats dosed with benzidine on the casein hydrolysate-tryptophan diet (Table V)

TABLE IV.—*Rats on Benzidine* (*Diet* 2).

| Survival time in days. | Abnormalities seen in organs. | | |
|---|---|---|---|
| | Bladder. | Liver. | Other organs. |
| 93 | — | Fatty degeneration. | — |
| 104 | None. | Fatty degeneration and regeneration of bile ducts. | — |
| 119 | Infected. | None. | — |
| 125 | Normal. | Hepatoma. | — |
| 126 | — | Fatty change and regeneration of bile ducts. | — |
| 127 | Infected. | None. | — |
| 150 | Early hyperplasia. | Fatty degeneration and regeneration of bile ducts. | — |
| 178 | Infected. | Fatty degeneration and bile duct carcinoma. | — |
| 199 | Normal. | Fatty generation and bile duct regeneration. | — |
| 224 | ,, | Fatty infiltration and bile duct regeneration. | — |

TABLE V.—*Rats on Benzidine and Tryptophan* (*Diet* 5).

| Survival time in days. | Abnormalities seen in organs. | |
|---|---|---|
| | Bladder. | Liver. |
| 202 | None. | Carcinoma. |
| 214 | — | — |
| 236 | None. | Early cholangioma. |
| 254 | — | Fatty degeneration and bile duct regeneration. |
| 305 | — | — |
| 331 | None. | None. |
| 424 | Early squamous hyperplasia. | Bile duct carcinoma. |

### EXPLANATION OF PLATE.

Carcinoma of the bladder induced by treating albino rats with acetamidofluorene for 488 days.

FIG. 2.—Malignant papilloma of the bladder. The tumour has projected into the viscus, and in some areas has undergone squamous changes. × 110.

FIG. 3.—Carcinoma infiltrating the submucosa. × 110.

FIG. 4.—Actively growing carcinoma, illustrating an area which had infiltrated the smooth muscle of the bladder wall. × 225.

2

3

4

Boyland, Harris and Horning.

lived more than 300 days none developed bladder tumours, but 2 of the group had liver tumours.

Of all the rats dosed with $\beta$-naphthylamine (Tables VI and VIII) 12 rats lived for over 700 days, but only one was seen with a papilloma of the bladder. With $\beta$-naphthylamine treatment and the casein hydrolysate-tryptophan diet (Table VII) 4 rats out of 12 were seen with hepatoma, whereas in the $\beta$-naphthylamine group on the casein diet only one hepatoma was seen out of the group of 9 rats which survived more than 100 days. The " casein hydrolysate-tryptophan diet " may therefore have increased the carcinogenic action of $\beta$-naphthylamine on the liver.

TABLE VI.—*Rats on $\beta$-Naphthylamine* (*Diet* 3).

| Survival time in days. | Abnormalities seen in organs. | | |
|---|---|---|---|
| | Bladder. | Liver. | Other organs. |
| 483 | — | — | — |
| 498 | — | — | — |
| 625 | None. | None. | — |
| 707 | Papilloma. | Fatty infiltration. | Early bronchogenic carcinoma. |
| 735 | None. | None. | — |
| 735 | None. | Slight fatty degeneration. | Lung carcinoma. Adrenal medulla haemorrhagic. |
| 769 | Slight proliferation of epithelium. | Hepatoma. | — |
| 770 | None. | Fatty degeneration. | Thyroid distended with colloid. |
| 925 | — | — | — |

TABLE VII.—*Rats on $\beta$-Naphthylamine and Tryptophan* (*Diet* 6).

| Survival time in days. | Abnormalities in organs. | | |
|---|---|---|---|
| | Bladder. | Liver. | Other organs. |
| 558 | None. | Hepatoma. | — |
| 571 | Some squamous metaplasia. | None. | Infected lung. |
| 605 | None. | ,, | Abscess in lung. |
| 658 | ,, | ,, | Bronchogenic carcinoma. |
| 662 | ,, | Hepatoma. | ,, ,, |
| 720 | ,, | — | Subcutaneous fibroma. |
| 782 | ,, | Hepatoma. | — |
| 782 | ,, | None. | Many acini in thyroid distended with colloid. |
| 799 | ,, | ,, | — |
| 841 | ,, | ,, | — |
| 943 | ,, | Hepatoma. | — |

The results with acetamidofluorene suggest that the casein hydrolysate-tryptophan diet protected the liver from the action of the carcinogen. Such a protective effect has been seen with antithyroid drugs (Paschkis, Cantarow and Stasney, 1948) and with thyroidectomy, (Bielschowsky and Hall, 1953). Since the effect of the tryptophan diet might have been due to an antithyroid action the effect of the diet on the thyroid glands of rats was examined. The results (Table VIII) show that the casein hydrolysate-tryptophan diet produced some enlargement of the thyroid gland, but thiouracil caused much greater enlargement of the thyroid gland. These experiments (Table VIII) showed also that the rats on the casein hydrolysate-tryptophan grew very slowly, so that the protective effect on the liver might be analogous to that observed by restriction of food intake.

TABLE VIII.—*The Effect of Different Diets on Growth and Thyroid Weights of Rats.*

| Diet. | Duration in days. | Number of rats used. | Body weight of rats (g.). Commencement of experiment. | Body weight of rats (g.). End of experiment. | Thyroid (mg. per 100 g. body wt.). Weight in mg./100 g. body weight. | Thyroid (mg. per 100 g. body wt.). Range of weights. | P. |
|---|---|---|---|---|---|---|---|
| Control | 7 | 10 | 203·8 | 214·8 | 6·22 | 4·5– 7·5 | — |
| Diet 1 without acetamidofluorene | 14 | 5 | 94·8 | 157 | 7·36 | 5·2– 9·0 | — |
| | 18 | 5 | 94·8 | 158·2 | 6·76 | 4·2–10·1 | — |
| 2% tryptophan + acid hydrolysed casein | 7 | 9 | 208·9 | 188 | 7·2 | 5·3– 8·8 | 0·05 |
| Diet 2 without acetamidofluorene | 14 | 5 | 97·5 | 97·2 | 9·7 | 7·8–11·6 | 0·1 |
| | 18 | 5 | 97·5 | 107·4 | 8·7 | 5·6–10·5 | 0·5 |
| 2% tryptophan + Diet 1 without acetamidofluorene | 14 | 5 | 93·7 | 110·4 | 6·7 | 4·3– 9·5 | — |
| | 18 | 5 | 93·7 | 119·8 | 7·5 | 5·1–10·9 | — |
| 0·033% 4 methylthiouracil+Diet 1 without acetamidofluorene | 14 | 5 | 96·9 | 139·8 | 20·4 | 13·7–32·2 | — |
| | 18 | 5 | 96·9 | 146 | 25·9 | 16·5–38·7 | — |

P = Probability of the significance of the difference from the control series.

### DISCUSSION.

The results confirm the findings of Dunning, Curtis and Maun (1950) in showing that the replacement of casein of the diet of rats treated with acetamidofluorene with a casein hydrolysate and *dl*-tryptophan caused bladder tumours to develop. The cause of this change in the site of the tumour induction is not obvious. One possibility is that the casein hydrolysate with tryptophan reduced the carcinogenic action of the acetamidofluorene on the liver and auditory meatus so that the animals lived long enough for bladder tumours to be induced. This idea is supported by the fact that the mean duration of life was 506 days in the " tryptophan " group and only 237 days in the " normal diet " group, and that one rat killed after 488 days had no liver tumour and 2 others killed after 483 and 488 days appeared to have only early bile-duct carcinomata. All the rats on the normal diet were dead before any bladder tumours were found in the " tryptophan " group.

The complete absence of bladder tumours in rats (except for rats of the Copenhagen and AXC strains) treated with acetamidofluorene in this series and in other reports on the effect of this carcinogen on normal diets suggests that the " tryptophan " diet may have a positive action in causing bladder cancer. This is supported by the results of Dunning, Curtis and Maun (1950) in which 22 out of 23 rats dosed with acetamidofluorene and tryptophan developed bladder tumours, the mean induction time being 400 days when the diet contained 1·4 per cent tryptophan and only 320 days when the diet contained 4·3 per cent tryptophan.

The fact that six of the rats on the " tryptophan " diet with $\beta$-naphthylamine lived for over two years without developing bladder cancer would indicate that *dl*-tryptophan itself is not carcinogenic towards the bladder. On the hypothesis that *ortho*-aminophenolic compounds cause cancer of the bladder, tryptophan might be suspected because animals dosed with large amounts of tryptophan excrete 3-hydroxyanthranilic acid and hydroxykynurenine.

The effect of the tryptophan in modifying the carcinogenic action of acetylaminofluorene may be due to—

(1) Summation of carcinogenic stimuli of acetamidofluorene metabolites and tryptophan metabolites.

(2) The tryptophan or tryptophan metabolites cause some hyperplasia of the bladder analogous to the effect of thiouracil in causing acetamidofluorene to induce tumours of the thyroid gland.

(3) Modification of the metabolism of acetamidofluorene so that more bladder acting carcinogen is formed.

(4) The casein hydrolysate-tryptophan diet might have an effect similar to that of a restricted diet or an antithyroid drug in protecting the liver from the carcinogenic action of acetamidofluorene.

Of these hypotheses the first seems unlikely because weak carcinogenic or inactive related substances generally inhibit carcinogenic action. The last two hypothetical mechanisms seem most probable and are being further investigated, but it is conceivable that acetamidofluorene itself has a direct carcinogenic action and the metabolites have a remote effect.

### SUMMARY.

Eight out of 10 rats dosed with acetamidofluorene in a diet in which the protein was replaced by an acid casein hydrolysate and 2 per cent *dl*-tryptophan developed tumours of the urinary bladder. Four rats treated with $\beta$-naphthylamine with the same diet developed liver tumours, while only one hepatoma occurred in the rats treated with $\beta$-naphthylamine on a normal diet.

We are indebted to Mr. Davidson Pratt of the Association of British Chemical Manufacturers, and to the British Drug Houses Ltd., for help in the supply of *dl*-tryptophan used in these experiments. This investigation has been supported by grants to the Royal Cancer Hospital and Chester Beatty Research Institute from the British Empire Cancer Campaign, the Jane Coffin Childs Memorial Fund for Medical Research, the Anna Fuller Fund, and the National Cancer Institute of the National Institutes of Health, U.S. Public Health Service.

### REFERENCES.

Bielschowsky, F. and Hall, W. H.—(1953) *Brit. J. Cancer*, **7**, 358.
Bonser, G. M.—(1943) *J. Path, Bact.*, **55**, 1.
*Idem*, Clayson, D. B. and Jull, J. W.—(1951) *Lancet*, ii, 286.
*Iidem* and Pyrah, L. N.—(1952), *Brit. J. Cancer*, **6**, 412.

BOYLAND, E. WALLACE, D. M. AND WILLIAMS, D. C.—(1954), *Biochem. J.*, **56**, xxix.
*Idem* AND WEIGERT, F.—(1947), *Brit. Med. Bull.*, **4**, 354.
DOBRINER, K., HOFMAN, K. AND RHOADS, C. P.—(1941) *Science*, **93**, 600.
DUNNING, W. F., CURTIS, M. R. AND MAUN, M. E.—(1950) *Cancer, Res.*, **10**, 454.
HUEPER, W. C.—(1938) *Arch. Path.*, **25**, 856.
*Idem* AND WILEY, F. H. AND WOLFE, H. D.—(1938) *J. industr. Hyg.*, **20**, 46.
KNOX, W. E. AND MEHLER, A. H.—(1951) *Science*, **113**, 237.
MANSON, L. A. AND YOUNG, L.—(1950) *Biochem. J.*, **47**, 170.
PASCHKIS, K. E., CANTAROW, A. AND STASNEY, J.–(1948) *Cancer Res.*, **8**, 257.
WALPOLE, A. L., WILLIAMS, H. H. C. AND ROBERTS, D. C.—(1952) *Brit. J. industr. Med.*, **9**, 255.
WILEY, F. H.—(1938) *J. biol. Chem.* **124**, 627.